# Mutations in *TGFbeta-RII *and *BAX *mediate tumor progression in the later stages of colorectal cancer with microsatellite instability

**DOI:** 10.1186/1471-2407-10-303

**Published:** 2010-06-18

**Authors:** Masakazu Yashiro, Kosei Hirakawa, C Richard Boland

**Affiliations:** 1 Department of Surgical Oncology, Osaka City University Graduate School of Medicine, Osaka, Japan; 2 Oncology Institute of Geriatrics and Medical Science, Osaka City University Graduate School of Medicine, Osaka, Japan; 3 Division of Gastroenterology, Baylor University Medical Center, Dallas, Texas, USA

## Abstract

**Background:**

Microsatellite instability (MSI) occurs in 15% of colorectal cancers (CRC). The genetic targets for mutation in the MSI phenotype include somatic mutations in the *transforming growth factor beta receptor typeII (TGFbetaRII), BAX, hMSH3 *and *hMSH6*. It is not clear how mutations of these genes mediate tumor progression in the MSI pathway, and the temporal sequence of these mutations remains uncertain. In this study, early stage CRCs were examined for frameshift mutations in these target genes, and compared with late stage tumors and CRC cell lines.

**Methods:**

We investigated 6 CRC cell lines and 71 sporadic CRCs, including 61 early stage cancers and 10 late stage cancers. Mutations of repetitive mononucleotide tracts in the coding regions of *TGFbetaRII, BAX, hMSH3, hMSH6, IGFIIR *and *Fas antigen *were identified by direct sequencing.

**Results:**

Thirteen (18.3%) of 71 CRC, including 9/61 (14.7%) early stage cancers and 4/10 (40%) late stage cancers, were identified as MSI and analyzed for frameshift mutations. No mutation in the target genes was observed in any of the 9 early stage MSI CRCs. In contrast, frameshift mutations of *TGFbetaRII, BAX, hMSH3 *and *hMSH6 *were present in 3/4 late stage MSI tumors. There is a statistical association (p = 0.014) between mutation in any one gene and tumor stage.

**Conclusions:**

*TGFbetaRII, BAX, hMSH3 *and *hMSH6 *mutations are relatively late events in the genesis of MSI CRCs. The frameshift mutations in these target genes might mediate progression from early to late stage cancer, rather than mediating the adenoma to carcinoma transition.

## Background

Microsatellite instability (MSI) is present in 15% of colorectal carcinomas (CRCs)[1]. Inactivation of the DNA mismatch repair (MMR) system leads to widespread somatic mutations at microsatellite loci. MSI tumors have been found to display microsatellite alterations not only in introns but also in coding exons. Genetic targets of this type of genomic instability include the *transforming growth factor β receptor type II *(*TGFβRII*), *insulin-like growth factor II receptor *(*IGFIIR*), *BAX*, *Fas antigen, hMSH3 *and *hMSH6*, all of which contain mononucleotide repeats in coding sequences [2,3]. Although it has been reported that MSI in introns is an early event in sporadic colorectal carcinogenesis [4,5], it is not known how mutations of these target genes are involved in tumor progression along the MSI pathway [2]. This study examined whether the frequency frameshift mutations in mononucleotide repeat regions within exons increases with stage in microsatellite unstable colorectal cancer. "Early stage cancer" was defined as intramucosal carcinoma, either carcinoma in situ or invasive cancer confined within the submucosa. For this study, "late stage cancer" was defined as any cancer invading the muscularis propria or serosa. Molecular events in early CRCs have not been well elucidated because of the limited availability of these cancers for detailed studies [5]. In this study, early stage MSI CRCs were examined for somatic frameshift mutations in the *TGFβRII*, *IGFIIR *and *BAX*, *hMSH3 *and *hMSH6 *genes, and compared with CRCS at more advanced stages and CRC cell lines.

## Methods

### Cell lines

Six CRC cell lines: HCT116, LoVo, SW480, LS174t, DLD1 and HT29, were obtained from the American Type Culture Collection (Rockville, MD), and maintained in tissue culture containing 10% fetal calf serum (GIBCO) at 37°C. LoVo, DLD1, SW480 and LS174t were derived from late stage colorectal cancer: LoVo [6] and DLD1 [7] were derived from Dukes' C tumor, and SW480 [8] and LS174t [9] were from Dukes' B tumor. Tumor stage of HCT116 [10] and HT29 [11] were not informative.

### Tumor tissues

Seventy-one CRCs, including 61 early stage cancers and 10 late stage cancers, were collected for analysis. The histopathological diagnosis was determined according to the classification by the World Health Organization (WHO) [12] or by the General Rules of the Japanese Research Society for Cancer of Colon and Rectum [13]. Lymph node status was not taken into account. The tumor tissues were microdissected from formalin-fixed, paraffin-embedded tissues sections as previously described [14]. Normal tissues were obtained from histologically normal mucosa or normal lymph nodes of the same patients. This study was approved by the Osaka City University ethics committee. Informed consent was obtained from all patients.

### DNA extraction

Genomic DNA from the cell lines was extracted with phenol-chloroform. Genomic DNA from tumor tissues was isolated using Proteinase K (Sigma, St. Louis, MO) at a final concentration of 100 μg/ml and were incubated for 5 h at 55°C [14].

### Identification of MSI

DNA was amplified by polymerase chain reaction (PCR) using ^32 ^P-end-labeled primers at microsatellite loci linked to the *hMSH2 *locus on 2p16 (*CA-5 *and *D2S123*), the *hMLH1 *locus on 3p23-21.3 (*D3S1611 *and *D3S1561*), the APC/MCC locus on 5q21 (*D5S107 *and *D5S346*), the p53 locus on 17p13 (*D17S513 *and *p53 *intron 1), and the *DCC/SMAD4 *locus on 18q21.3 (*D18S35A *and *18qDCC-TA*) [14]. We utilized stringent criteria for the determination of MSI from a National Cancer Institute workshop [15]. MSI-high (referred to as MSI) was defined by a novel band shift or allele at >20% of the microsatellite loci tested when compared to non-neoplastic tissue from the same patient. Our assay for MSI was based on PCR amplification of a panel of 10 microsatellite primer pairs linked to tumor suppressor genes. In addition, poly (T)_27 _mononucleotide repeats in the noncoding region of *β-catenin *exon 16 was performed using the following primer sets: 5'-GGTACTGACTTTGCTTGCTT-3' and 5'-ACTTAACACTACGAGAGACT-3'. In each case, one of the primer pairs was end-labeled with [γ-^32^P]ATP and subjected to PCR. The resulting PCR products were analyzed for MSI by electrophoresis in 8% polyacrylamide gel containing 7.5 M urea and 0.5× TBE.

### Frameshift mutation analysis

Mutations of repetitive mononucleotide tracts in the coding regions of *TGFβRII*, *IGFIIR*, *BAX*, *hMSH3*, *hMSH6 *and *Fas antigen *were identified using the following primer sets; 5'-CTTTATTCTGGAAGATGCTGC-3' and 5'-GAAGAAAGTCTCACCAGGC-3' for the poly(A)_10 _tract of *TGFβRII*, 5'-GCAGGTCTCCTGACTCAGAA-3' and 5'-GAAGAAGATGGCTGTGGAGC-3' for the poly(G)_8 _tract of *IGFIIR*, 5'-ATCCAGGATCGAGCAGGGCG-3' and 5'-ACTCGCTCAGCTTCTTGGTG-3' for the poly(G)_8 _tract of *BAX*, 5'-AGCTGGATGATGCTGTAAAT-3' and 5'-TTCCTCACCTGCAAAGTACT-3' for the poly(A)_8 _tract of *hMSH3*, 5'-CGCCCAGTAATTCTGTTG-3' and 5'-CATTTTCCTGCTCCTCTTC-3' for the poly(C)_8 _tract of *hMSH6*, and 5'-ACCCGGACCCAGAATACCAA-3' and 5'-GCAAGGGTCACAGTGTTCAC-3' for the poly(T)_7 _tract of *Fas antigen*. To confirm mutations observed as band shifts, DNA was excised from the gel, purified with the Microspin columns (Pharmacia Biotec, Inc., Uppsala, Sweden), and reamplified with the same primer. The amplified products were sequenced by the dideoxychain termination method, with the AmpliCycle sequencing kit (Perkin-Elmer, Branchburg, NJ).

## Results

### Frameshift mutations in CRC cell lines

**Figure 1:**
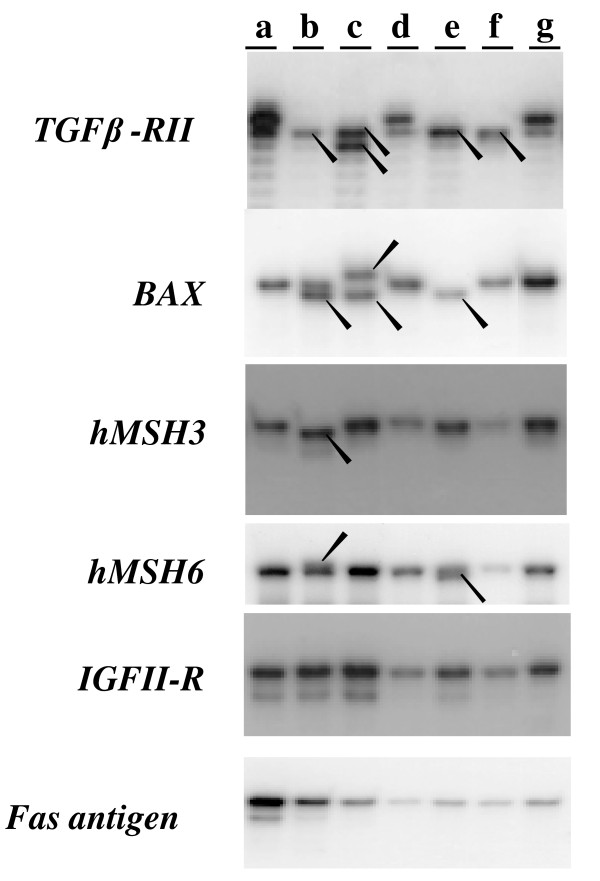
**Frameshift mutations in CRC cell lines**. Lane a: products from normal control DNA. The products of MSI cell lines are shown in Lanes b (HCT116), c (LoVo), e (LS174-T) and f (DLD1). Products of non-MSI cell lines are shown in Lanes d (SW480) and g (HT29). The genes are indicated at the left. Arrowheads indicate frameshift mutations in cell lines.

It has been reported that HCT116, LoVo, LS174t and DLD1 have the MSI phenotype, whereas SW480 and HT29 do not [16]. In this study, frameshift mutations in the (A)_10 _tract of *TGFβRII *were found in all 4 MMR-deficient cell lines: HCT116, LoVo, LS174t and DLD1. All cell lines have only mutant alleles of TGF*β*RII, either (A)_9 _or (A)_8_. Microsatellite alterations in the (G)_8 _tract of *BAX *were present in HCT116, LoVo and LS174t. LoVo and LS174t cells had only (G)_9 _and (G)_7 _mutant BAX alleles; HCT116 had one (G)_7 _mutant allele and one (G)_8_wild-type allele. Deletions in the (A)_8 _tract of the coding sequence of *hMSH3 *were present in HCT116. Insertion and deletion mutations in the (C)_8 _tract of *hMSH6 *were detected in HCT116 and LS174t, respectively. In contrast, the MMR-proficient cell lines SW480 and HT29 demonstrated microsatellite stability, and did not have mutations in *TGFβRII, BAX*, *hMSH3 *or *hMSH6*. No alterations in the (G)_8 _tract of *IGFIIR *or the (T)_7 _tract of *Fas antigen *were found in any of the cell lines examined (Figure 1).

### Frequencies of frameshift mutations in early and late stage sporadic MSI CRCs

**Figure 2:**
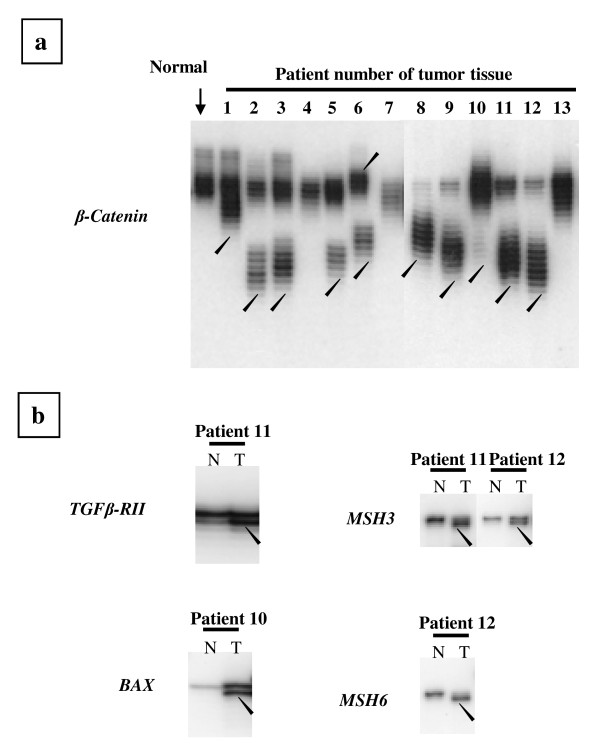
**Frameshift mutations in CRCs with MSI**. The genes are indicated at the left. Arrowheads indicate 1 bp deletion mutations in tumors. T and N refer to DNA from tumors and corresponding normal tissues, respectively.

**Figure 3:**
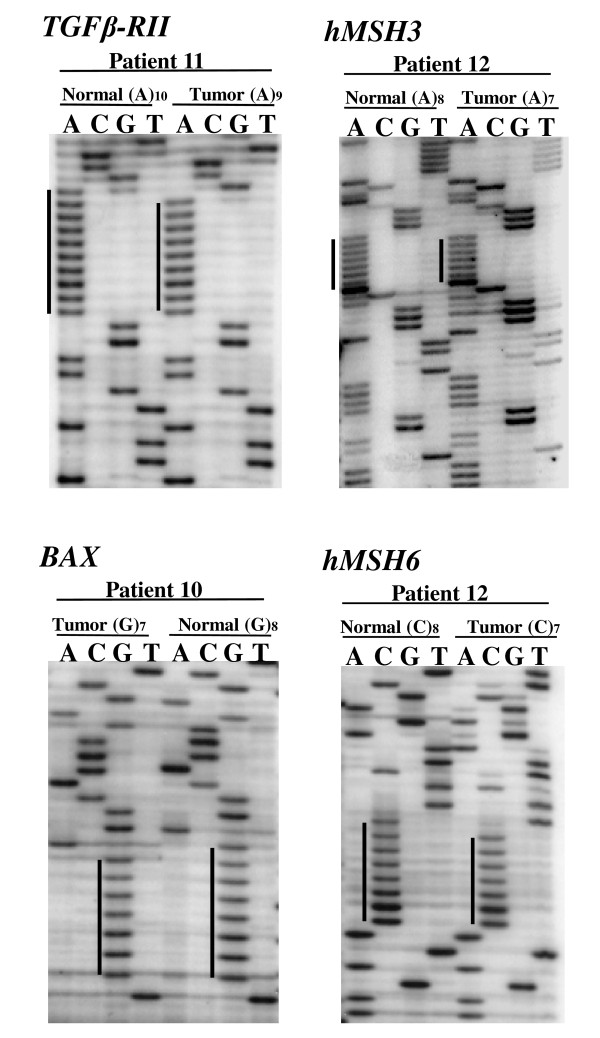
**Sequence analysis of frameshift mutations in late stage CRCs**. Single one bp deletions in the mononucleotide repeats were found in each case.

Thirteen of 71(18.3%) sporadic CRCs, including 9/61 (14.7%) early stage CRCs and 4/10 (40%) late stage cancers were identified as MSI tumors (Table 1), and these tumors were analyzed for frameshift mutations. Mutations in the noncoding region of *β-catenin*, which contains a (T)_27 _sequence, were present in 7/9 MSI early stage CRCs (patients 1-9) and 3/4 MSI-H late stage CRCs (patients 10-13) (Figure 2A). No frameshift mutations in the target genes were present in any MSI early stage CRCs. In contrast, frameshift mutations of *TGFβRII*, *BAX*, *hMSH3 *and *hMSH6 *were present in 3/4 late stage CRCs (Table 1). A significant difference (p = 0.014) was found in the frequency of frameshift mutations between early stage and. late stage (Table 2). *TGFβRII *and *BAX *mutations were found in the late stage tumors of patients 11 and 10, respectively (Figure 2B). *hMSH3 *mutations were present in patients 11 and 12, and an *hMSH6 *mutation was found in patient 12. Each mutation was found to be a 1 bp deletion in the polynucleotide tract (Figure 3). No frameshift mutations in *IGFIIR *or *Fas antigen *were found in any CRC.

**Table 1:** Frameshift mutations in MSI CRCs

Patient number	Stage	Depth of invasion^*a*^	Tumor size	Location^*b*^	MSI	Mutations in mononucleotide repeat tracts					
						
			(mm)			*TGFβRII*	*BAX*	*hMSH3*	*hMSH6*	*IGFIIR*	*Fas antigen*
1	Early	m	4	A	2/6	- ^*c*^	-	NI^*d*^	-	-	-
2	Early	m	4	A	2/8	-	-	NI	-	-	-
3	Early	m	3	S	3/8	-	-	-	-	-	-
4	Early	sm	8	R	3/8	-	-	-	-	-	-
5	Early	sm	14	A	2/5	-	-	NI	-	-	-
6	Early	sm	8	T	3/8	-	-	-	-	-	-
7	Early	sm	4	D	3/10	-	-	-	-	-	-
8	Early	sm	12	NI	2/8	-	-	NI	-	-	-
9	Early	sm	13	R	2/9	-	-	-	-	-	-
10	Late	mp	9	R	2/8	-	De	-	-	-	-
11	Late	mp	20	S	3/9	De ^*e*^	-	De	-	-	-
12	Late	mp	65	A	4/10	-	-	De	De	-	-
13	Late	mp	24	S	2/9	-	-	-	-	-	-

^*a*^m, carcinoma tissues were restricted to the mucosa; sm, the deepest carcinoma invasion was in the submucosa; mp, the deepest carcinoma penetration reached the muscularis propria.

^*b*^A, ascending colon; T, transverse colon; D descending colon; S, sigmoid colon; R, rectum.

^*c *^-, wild type.

^*d *^NI, not informative.

^*e *^De, deletion.

**Table 2:** Correlation between tumor stage and frameshift mutations.

	Frameshift mutation		
		
Tumor stage	positive	negative	p value
Early	0	9	
Late	3	1	0.014

Significance level of difference was determined using Fisher's exact test.

## Discussion

Although CRCs with the MSI phenotype evolve because of loss of DNA MMR activity, there is little consensus on the sequence and timing of target gene inactivation in the evolution of tumors that occur through this pathway. It has been reported that the appearance of MSI, at least at noncoding microsatellites, is an early event in Lynch syndrome CRCs [4,17], and that frameshift alterations in the functionally critical target genes may occur at an early stage in these tumors [18-20]. *TGFβRII *frameshift mutations are present in 80-90% of MSI CRCs, and it has been reported that these occur at the adenoma-carcinoma transition in MSI adenomas [21,22]. The precise timing of the onset of MSI, and the occurrence of mutations at other genetic targets, have not been confidently determined.

In this study, we examined the timing of frameshift mutations of *TGFβRII*, *IGFIIR*, *BAX*, *hMSH3*, *hMSH6 *and *Fas antigen *in 9 early stage and 4 late stage MSI CRCs. Interestingly, no mutations were identified in any early stage MSI-H CRC at *TGFβRII*, *BAX, hMSH3 *or *hMSH6 *, but these were relatively frequent events in late stage MSI CRCs, and in the MSI CRC cell lines. There is a statistical association between mutation in any one gene and tumor stage. The frequency of frameshift mutations in mononucleotide repeat regions within exons might increase with stage in microsatellite unstable colorectal cancer. Our findings suggest that *TGFβRII*, *BAX*, *hMSH3 *and *hMSH6 *frameshift mutations are relatively later stage events in tumor progression for sporadic CRC with MSI. It has been reported that *TGFβRII *mutations are infrequent in the early stages of sporadic gastric cancers with MSI [23], but these occur more frequently in gastric tumors that are larger [24]. It has been reported that the loss of *TGFβRII *in intestinal epithelial cells promotes the invasion and malignant transformation of tumors initiated by *APC *mutations [25]. However, few studies have clarified how mutations of the target genes we have studied mediate tumor development in the MSI pathway. These findings suggested that the mutations of *TGFβRII*, *BAX*, *hMSH3*, and *hMSH6 *might play an important role in cancer progression from early to late stage tumors, rather than early in carcinogenesis. However, there still may be additional, currently unappreciated genes or DNA sequences involved in the process [18,26-28]. Interestingly, no microsatellite mutations in *IGFIIR *and *Fas antigen *were found in any cell line or in any CRC. It has also been reported that no mutations in the *IGFIIR *genes were found in 27 MSI-H gastric tumors [29]. These findings suggest that mononucleotide repeat of *IGFIIR *and *Fas antigen *might be unimportant as target genes in MMR-deficient CRCs. A limitation of our study is the small number of tumor samples, while a significant difference between the early stage cancer and late stage cancers was recognized. Large numbers of patients with MSI cancer might be necessary in the future to conclude the correlation between tumor stage and frameshift mutations in target gene.

## Conclusions

Our data confirm that MSI can be found very early in the evolution of a colorectal tumor with MSI, we propose that not all sequence alterations are equivalent, and that mutations in the coding microsatellites of *TGFβRII *and *BAX *mediate progression from early to late stage CRC with MSI. These finding raise interesting possibilities for diagnosis as well as treatment.

## Competing interests

The authors declare that they have no competing interests.

## Authors' contributions

MY participated in the study design, and carried out PCR experiments, sequencing analysis, interpretation of data, and paper preparation. KH carried out data analysis and interpretation. CRB carried out data analysis, interpretation, and paper preparation. All authors have read and approved of the final manuscript.

## Pre-publication history

The pre-publication history for this paper can be accessed here:

http://www.biomedcentral.com/1471-2407/10/303/prepub
